# Abnormal synergistic gait mitigation in acute stroke using an innovative ankle–knee–hip interlimb humanoid robot: a preliminary randomized controlled trial

**DOI:** 10.1038/s41598-021-01959-z

**Published:** 2021-11-24

**Authors:** Chanhee Park, Mooyeon Oh-Park, Amy Bialek, Kathleen Friel, Dylan Edwards, Joshua Sung H. You

**Affiliations:** 1 Sports Movement Artificial-Intelligence Robotics Technology (SMART) Institute, Department of Physical Therapy, Yonsei University, 1 Yonseidae-gil, Wonju, Gangwon-do 26493 Republic of Korea; 2 Department of Physical Therapy, Yonsei University, Wonju, Republic of Korea; 3 Burke Rehabilitation Hospital, White Plains, NY USA; 4 Albert Einstein College of Medicine, Montefiore Health System, White Plains, NY USA; 5 Burke Neurological Institute, White Plains, NY USA; 6 Moss Rehabilitation, Elkins Park, PA USA; 7 Edith Cowan University, Joondalup, Australia

**Keywords:** Diseases, Medical research, Neurology

## Abstract

Abnormal spasticity and associated synergistic patterns are the most common neuromuscular impairments affecting ankle–knee–hip interlimb coordinated gait kinematics and kinetics in patients with hemiparetic stroke. Although patients with hemiparetic stroke undergo various treatments to improve gait and movement, it remains unknown how spasticity and associated synergistic patterns change after robot-assisted and conventional treatment. We developed an innovative ankle–knee–hip interlimb coordinated humanoid robot (ICT) to mitigate abnormal spasticity and synergistic patterns. The objective of the preliminary clinical trial was to compare the effects of ICT combined with conventional physical therapy (ICT-C) and conventional physical therapy and gait training (CPT-G) on abnormal spasticity and synergistic gait patterns in 20 patients with acute hemiparesis. We performed secondary analyses aimed at elucidating the biomechanical effects of Walkbot ICT on kinematic (spatiotemporal parameters and angles) and kinetic (active force, resistive force, and stiffness) gait parameters before and after ICT in the ICT-C group. The intervention for this group comprised 60-min conventional physical therapy plus 30-min robot-assisted training, 7 days/week, for 2 weeks. Significant biomechanical effects in knee joint kinematics; hip, knee, and ankle active forces; hip, knee, and ankle resistive forces; and hip, knee, and ankle stiffness were associated with ICT-C. Our novel findings provide promising evidence for conventional therapy supplemented by robot-assisted therapy for abnormal spasticity, synergistic, and altered biomechanical gait impairments in patients in the acute post-stroke recovery phase.

Trial Registration: Clinical Trials.gov identifier NCT03554642 (14/01/2020).

## Introduction

The advanced research and development of innovative Robotic-Assisted Gait Training (RAGT) systems in the field of robotic science have recently provided powerful and promising progress and, hence, hope for millions of patients with synergistic hemiparetic gait after stroke^1^. Based on the contemporary task-oriented locomotor learning theory, current stroke RAGT paradigms involve two commonly utilized systems (the Lokomat hip-knee exoskeletal static RAGT, overground RAGT and G-EO end-effector RAGT) to mitigate the different aspects of abnormal synergetic gait patterns^1^. The hip-knee exoskeletal static RAGT uses a top-down biomechanical model^2^ to focus on the hip and knee joint movements. The end-effector RAGT uses the bottom-up model, emphasizing the ankle joint movement, which is often supported by a foot plate during locomotor retraining^3–5^. The overground wearable RAGT (Ekso Ekso Bionics, Richmond, CA, USA) uses the bottom-up model, which actuates movements of the hip and knee joints only for over-ground gait training in stroke^6,7^. While both exoskeletal, end-effector, and wearable RAGT systems have gained tangible improvements in gait function and the associated biomechanical characteristics in patients with stroke ^3,4,6,7^, the important issue and underlying synergetic gait problem remains unsolved and warrants further research and development^3,8^. The synergetic hemiparetic gait involves the loss of selective ankle–knee–hip joint movement coordination, which is associated with abnormal spasticity, stiffness, and synergy due to cortical disinhibition post-stroke^8–11^. Clinically, the synergistic hemiparetic gait is classified as flexor and extensor and concurrently manifests with increased spasticity and associated stiffness. The extensor synergetic gait is characterized by more increased ankle plantarflexion, knee hyperextension, and hip internal rotation and extension along with a compensatory circumduction gait when compared to normal controls^12–15^. Specifically, the lack of open chain dorsiflexion in the terminal stance results from dorsiflexor muscle weakness and spastic plantarflexors’ activity^13,16^. Knee hyperextension in the stance phase is observed to be compensating for the insufficient closed-chain dorsiflexion so that the tibia rotates anteriorly, pivoting around the talocrural joint axis^16^. The quadriceps muscles are further weakened and cannot support the knee and ankle during the stance phase^17^. Hip circumduction gait is a compensatory pattern for iliopsoas and gluteus muscle weakness (50%) and the improper forward moment and longer level arm for foot clearance^16^. On the other hand, the flexor synergetic gait is characterized by more increased external rotation, abduction, and flexion of the hip (2.1°), flexion of the knee (19°), flexion (10°), and inversion of the ankle than normal controls^18,19^. Insufficient plantarflexion occurs due to more gastrocnemius weakness and eccentric motor control to advance the foot anteriorly during the terminal stance and early swing phases than normal controls^12,13,18,19^. The knee hyperflexion is associated with more quadriceps muscle weakness, hip hyperflexion (6.5°), and external rotation (0.5°) due to the knee flexion during the swing phase when compared to normal controls^14,20–23^, ultimately leading to gait dysfunction in 85% of hemiparetic stroke population^15^. Therefore, the present rationale for the robot-assisted training was to ‘break the abnormal ankle–knee–hip synergy’ or improve selective ankle–knee–hip locomotor coordination in gait rehabilitation after stroke, rather than focus on the amelioration of the hip-knee or ankle joint synergy^3,24,25^. In an extensive systematic review of the current RAGT studies, patients with hemiparetic stroke were reported to exhibit an inherent abnormal synergistic gait impairment, particularly in the ankle joint plantarflexor synergy even after intensive RAGT. However, the overall gait function was enhanced^9,26^. Such unresolved abnormal ankle synergy may have stemmed from the insufficient locomotor coordination of ankle–knee–hip movement control in the currently used RAGT and end-effector RAGT systems^27,28^. This scientific evidence corroborates the reported superior effects of RAGT with ankle–knee–hip interlimb locomotor coordination control on volitional locomotor movement with “normal synergy” and motor control when compared to RAGT without ankle joint control (only knee-hip)^24,29^. As such, stroke robotic rehabilitation clearly mandates for more effective and sustainable ankle–knee–hip interlimb coordinated locomotor control to intervene on the synergistic gait impairment.

To overcome such shortcomings of the current exoskeletal (hip-knee control only) and end effector (ankle control only) models, we have developed an innovative ankle–knee–hip interlimb coordinated humanoid robot training (ICT) system (Walkbot, P&S Mechanics, Seoul, Republic of Korea). The ICT system is primarily designed to create the optimal ankle–knee–hip interlimb coordinated locomotor movement, thereby mitigating such underlying abnormal synergistic gait impairment in stroke rehabilitation^26,30,31^. This robotic system can detect the patient’s current gait characteristics, such as the amount of participation or use in terms of active joint, angular displacement excursion, active force/torque, and active weight-bearing center of pressure. The ICT system calculates real-time ankle–knee–hip joint angles, joint moment, and muscle forces using a musculoskeletal anthropometry model, including bone lengths, joints, inertial parameters tendon attachments. It can be personalized to reflect subject-specific anatomic morphology^26,30^*.* Building on the contemporary motor learning theory of task specificity, the ICT system allows accurate proprioceptive, kinematic, and kinetic guidance and real-time motivational feedback concerning ankle–knee–hip kinematics and kinetics^32^. Importantly, ICT system enables clinicians to provide variable error practice and high-intensity, repetitive, task-specific, and interactive exercises of the paretic lower limb^26,33^. Recent ICT system empirical and clinical studies demonstrated excellent validity (*R*^2^ = 0.86)^34^ and promising clinical improvements in balance and gait function (63.4%, 14.2%, and 15%) and biomechanical characteristics (kinematics; 29.8%, 15.8% and 66.6%) in patients with hemiparetic stroke, spinal cord injury, and cerebral palsy, respectively^24,26,31,35–37^*.*

Based on the conceptual framework of the ankle–knee–hip interlimb locomotor coordination on synergy control, the present research has two specific aims. The primary purpose was to ascertain the therapeutic effects of ankle–knee–hip Interlimb Coordinated robotic Training combined with Conventional physical therapy (ICT-C; 30 min of ICT in addition to 60 min of physical therapy) on abnormal lower-extremity synergistic pattern, which was determined using the standardized Fugl-Meyer Assessment of Lower Extremity (FMA-LE), when compared to those of Conventional Physical Therapy and Gait training (CPT-G; 30 min of gait training + 60 min of physical therapy) in patients with acute hemiparetic stroke. The secondary purpose aimed to investigate the biomechanical changes associated with Walkbot ICT during acute rehabilitation, on kinematic (spatiotemporal and angles) and kinetic (active force, resistive force, and stiffness) gait parameters, and to investigate the ICT-C on spasticity which was determined using the Modified Ashworth Scale (MAS), compared to CPT-G in patients with acute hemiparetic stroke. Correspondingly, our primary hypothesis was that there would be differences in spasticity and abnormal lower-extremity synergistic pattern between the ICT-C and CPT-G. Our secondary hypothesis was that there would be significant differences in the kinematic and kinetic gait parameter data between pre-and post-ICT intervention.

## Materials and methods

The present clinical research goals were twofold: The prirmairy goal was to determine the therapeutic effects of ICT-C on abnormal lower-extremity synergistic pattern, which was determined using the standardized FMA-LE, when compared to those of CPT-G in patients with acute hemiparetic stroke. The secondary goal was to examine the biomechanical changes associated with Walkbot ICT during acute rehabilitation, on kinematic (spatiotemporal and angles) and kinetic (active force, resistive force, and stiffness) gait parameters, and the ICT-C on spasticity using the MAS, compared to CPT-G in patients with acute hemiparetic stroke.

### Patients

A convenience sample of 20 patients with acute hemiparetic stroke (mean age 73.0 ± 12.72 years, 12 women) were enrolled as inpatients at the Burke rehabilitation hospital, New York, United states. The Albert Einstein college of medicine institutional review board and the ethical committee (No. 2018-9283) approved the experimental study protocol. After the patients were recruited via bulletin board notices within the hospital, initial screening was conducted to determine whether the potential patients met the inclusion criteria. Informed consent was obtained from all the patients before participation. This study was conducted by the relevant guidelines/regulations and confirmed that informed consent was obtained from all patients and/or their legal guardians. The study was conducted in accordance with the Declaration of Helsinki. The inclusion criteria were as follows: (1) acute cortical/subcortical ischemic stroke (2 weeks post-stroke onset); (2) age between 18 and 99 years; (3) first clinical stroke presentation or prior stroke with no residual deficits affecting ambulation; (4) ability to follow a two-step command; (5) Fugl-Meyer sensory score > 2; (6) suitability for gait training as assessed clinically (ability to ambulate at least one step with a device/assistance); (7) height 132–200 cm; (8) hip-knee joint length 33–48 cm; and (9) knee joint-to-foot length, 33–48 cm. The exclusion criteria were as follows: (1) cerebellar/brainstem stroke, (2) body weight > 135 kg, (3) uncontrolled hypertension (stage 2) with blood pressure > 160/100 mmHg; (4) cardiopulmonary impairments that can affect the ambulation test; (5) integumentary impairment such as skin breakdown or bedsores around the suspension belt loading region; (6) relevant and persistent mental illness; (7) lower-extremity fixed contracture or deformity; (8) bone instability (nonconsolidated fractures, unstable spinal column, or severe osteoporosis necessitating treatment with bisphosphonates), (9) other neurodegenerative disorders (amyotrophic lateral sclerosis, Parkinson’s disease); (10) MAS score > 3 in the affected leg; (11) relevant back or leg pain resulting in an inability to tolerate movement; (12) decreased sensation impairing the ability to perceive whether the device is properly fitted, and (13) aphasia sufficient to prevent the ability to communicate discomfort. Table 1 shows inter-group comparisons of baseline demographics and clinical characteristics of the patients. The nonparametric chi-square test for categorical variables showed no significant differences in demographics or clinical characteristics between the groups.

**Table 1:** Demographic and clinical characteristics of the patients (N = 20).

Characteristics	CPT-G (n = 10)	ICT-C (n = 10)	*P*-value
**Age (years)**	70.60 ± 13.60	75.40 ± 11.21	0.749
**Sex**			
Male (%)	3 (30)	5 (50)	0.206
Female (%)	7 (70)	5 (50)	
**Type of stroke**			
Onset time (days)	13.20 ± 7.20	7.60 ± 4.95	0.232
Ischemic (%)	10 (100)	10 (100)	–
**Side of hemiplegia**			
Left (%)	6 (60)	6 (60)	0.513
Right (%)	4 (40)	4 (40)	

*CPT-G* conventional physical therapy and gait training, *ICT-C* ankle–knee–hip interlimb coordinated humanoid robot combined with conventional physical therapy.

### Experimental procedure

A preliminary, randomized, single-blind, experimental design was used in the present study. Coin flipping was used to assign patients to either the control or experimental group. A researcher generated the random allocation sequence, another researcher assigned patients to interventions, and a third-party blinded researcher assessed outcome measures. To remove experimental biases associated with the patients’ expectations, experimental information that could affect the patients was masked until the experiment was completed. A consistent experimental procedure was followed using intervention and standardized tests, including MAS, and FMA-LE clinical measurements for both CPT-G and ICT-C groups before and after the intervention. Additionally, biomechanical data including kinematic (e.g., angles), kinetic (e.g., active and resistive force), and resistive stiffness in hip, knee, and ankle joints were measured before and after ICT-C. The same investigators conducted all tests and interventions to improve the internal validity of the measurements.

### Hardware

The hardware comprised an actuator module, a control module, and a power module. The actuator module was rigidly attached to an exoskeleton and secured to the lower limbs using a velcro belt. The ICT system was rigidly secured to the patients’ upper body (i.e., chest) using adjustable belts.

### Actuator module

This module comprises two three-phase direct-current brushless motors, each providing output torque to the hip, knee, and ankle joints. The motors have a drive voltage of 24.0 V, a rated load current of 2.0 A, and a maximum thrust load of 3.8 N.

### Impedance control

The approach implemented for the ICT system was position-based impedance control^38^*.* Mechanical impedance can be treated as the relationship between the force exerted by the actuators and the resulting motion. As the mechanical impedance is viscoelastic, the restoring force is related to the deviation of the robot's reference trajectory and velocity. However, a dead-band and a limited threshold of angle deviation were introduced to allow the normal variability of the human gait pattern^39,40^. The robot would only intervene if the set level of trajectory deviation was exceeded.

The position-based impedance control law in joint space is given by

1 $${\varvec{u}} = \hat{\user2{F}}({\varvec{q}}){\varvec{a}}_{q} + \hat{\user2{u}}_{ext} + \hat{\user2{C}}({\varvec{q}},\dot{\user2{q}})\dot{\user2{q}} + \hat{\user2{f}}\user2{r}(\dot{\user2{q}}) + \hat{\user2{g}}({\varvec{q}})$$

where $\hat{\user2{u}}_{ext}$ is the estimated external torque from the reaction torque observer.

The estimation of external torque is based on inverse dynamics

2 $$\hat{\user2{u}}_{ext} = \frac{g}{s + g}\left( {{\varvec{u}}_{a} + {\varvec{g}}\widehat{{\varvec{F}}}({\varvec{q}})\dot{\user2{q}} + {\varvec{g}}\widehat{{\varvec{F}}}({\varvec{q}})\dot{\user2{q}} + {\varvec{g}}\widehat{{\varvec{F}}}({\varvec{q}})\dot{\user2{q}}} \right)$$

where $\frac{g}{s+g}$ is a lowpass filter and *g* is its cutoff frequency.

The acceleration term ***a***_*q*_ takes the following form:

3 $${\varvec{a}}_{q} = {\mathbf{q}}_{d} + {\varvec{K}}_{p} {\varvec{e}}_{Imp} + {\varvec{K}}\nu \dot{\user2{e}}_{Imp}$$

4 $${\varvec{e}}_{Imp} = {\varvec{q}}_{d} - \hat{\user2{u}}_{ext} {\varvec{Z}}d^{ - 1} - {\varvec{q}}$$

5 $$\dot{\user2{e}}_{Imp} = {\varvec{q}}_{d} - \hat{\user2{u}}_{ext} s{\varvec{Z}}d^{ - 1} - {\varvec{q}}$$

where ***q***_*d*_ denotes the desired position and ***e***_*Imp*,_ and ***e*˙**_*Imp*_ denote the impedance error and its first derivative. ***K***_*v*_ and ***K***_*p*_ ∈ ℜ^2×2^ denote the diagonal derivative and proportional controller gain matrices. ***Z***_*d*_ = ***F***_*d*_*s*^2^ + ***B***_*d*_*s* + ***K***_*d*_ denotes the desired impedance model.

**Figure 1:**
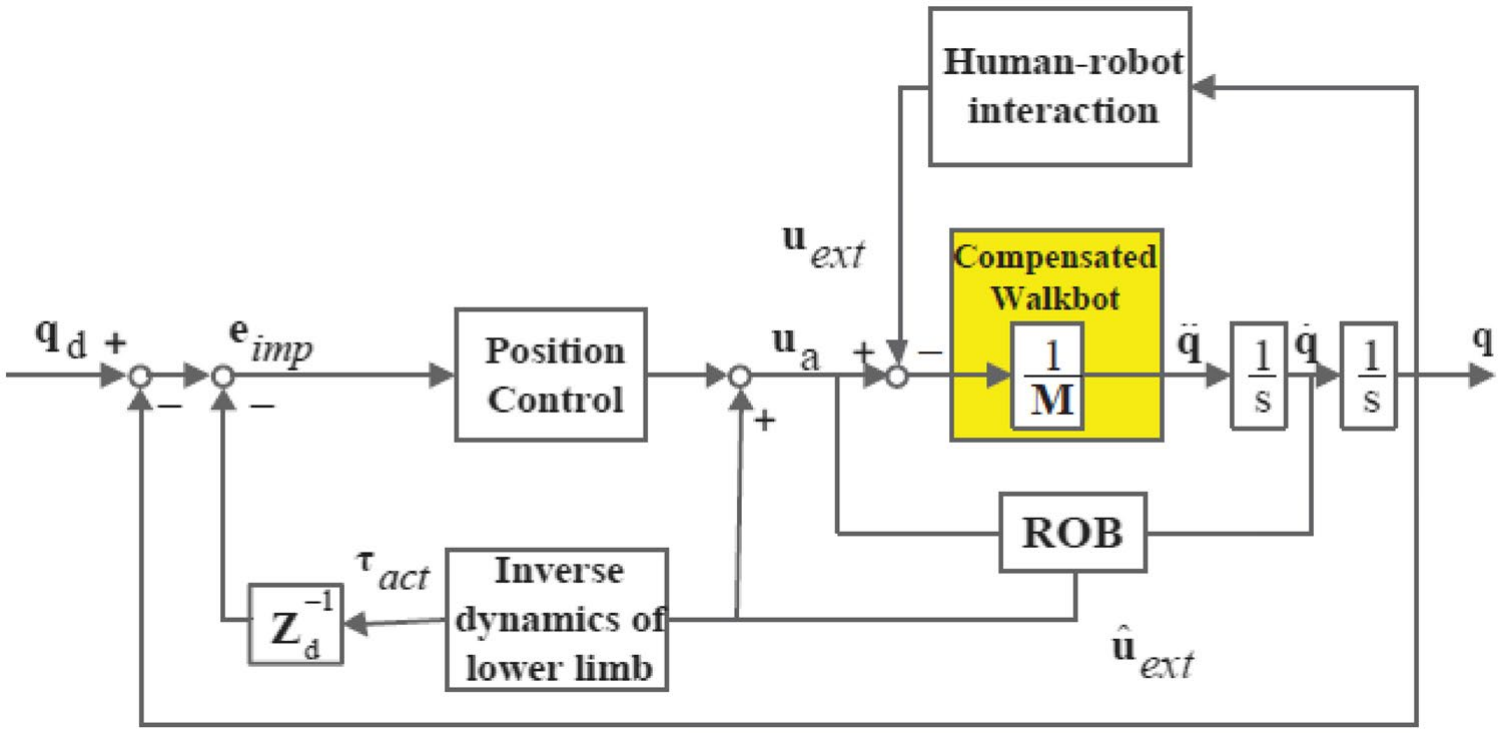
The control scheme of the position-based impedance control for gait rehabilitation. *ROB* reaction torque observer.

***F***_*d*_, ***B***_*d*_*s*, and ***K***_*d*_ ∈ ℜ^2×2^ are the desired mass matrix, damping matrix, and stiffness matrix. In (4) and (5), the estimated torque feedback resulted in deviations of reference angular position and velocity. The overall scheme of the force/torque sensorless position-based impedance control algorithm is shown in Fig. 1^38^. The value of mechanical impedance was manipulated by a therapist based on their experience, considering the patient’s movement capability and disability levels. By adjusting the virtual mechanical impedance parameters, the therapist could make the training more or less demanding for the patient. With a lower impedance, the patient needed to participate more actively to maintain a functional gait pattern. In practice, only ***K*** was adjusted by the therapist, and ***B*** would automatically adapt as a function of ***K***.

### Biomechanical measurements for kinematics, kinetics, and stiffness

The ICT system features a biomechanical function to achieve efficient walking based on the inverted pendulum model^38^. Biomechanical characteristics were determined using the kinematic and kinetic computing software (P&S Mechanics, Seoul, Korea) of the ICT system, which calculates the angular displacement and active and resistive hip, knee, and ankle joint forces and torques^38^. Kinematic and kinetic data were synchronously obtained from each of the five gait cycles in a steady-state, lasting > 5 min, from all patients before and after the intervention.

**Figure 2:**
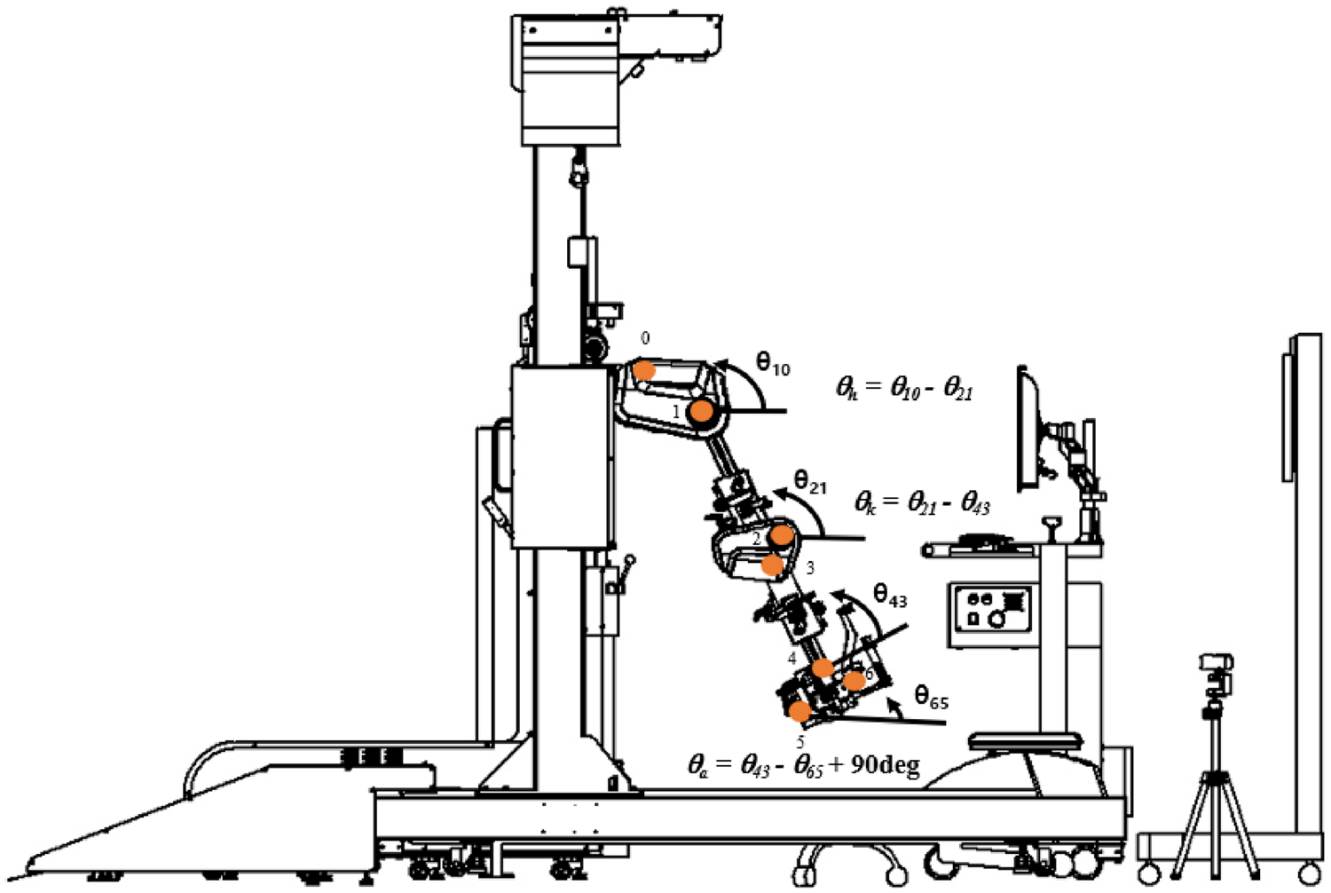
Lower-extremity kinematic joint angle calculation in ICT system. *ICT* innovative ankle–knee–hip interlimb coordinated humanoid robot.

Kinematic measurements encompassed the joint angle, angular velocity, and acceleration, which were then used to calculate the moment or torque associated with the body segment's active and resistive forces acting on the ankle, knee, and hip joints of the participant during walking. For example, when in full extension, the hip is defined as at 0° flexion. When the thigh moves in an anterior direction relative to the pelvis, the hip is defined as being in flexion (Fig. 2)^41^.

$${\text{Hip }}\;{\text{angle }} = \theta_{h} = \theta_{10} - \theta_{21}$$

If *θ*_*10*_ > *θ*_*21,*_ the hip is in flexion; if *θ*_*10*_ < *θ*_*21,*_ the hip is extended.

$${\text{Knee }}\;{\text{angle }} = \theta_{k} = \theta_{21} - \theta_{43}$$

$${\text{Ankle }}\;{\text{angle }} = \theta_{a} = \theta_{43} - \theta_{65} + { 9}0^\circ$$

The convention for the ankle was slightly different, in that 90° between the leg and the foot was the boundary between plantarflexion and dorsiflexion. If *θ*_*a*_ is positive, the foot is in plantar flexion; if *θ*_*a*_ is negative, it is in dorsiflexion. Kinematic data were collected using a built-in potentiometer in the Walkbot system with a sample rate of 36 Hz.

Kinetic measurements included active and resistive forces and torques of the body segment acting on the hip joint during robotic interactive gait training. With the thigh lever arm acting on the robot system, the recorded force data can be converted into hip joint torques acting between the ICT system and the participant’s leg. The ankle–knee–hip joint torque data were collected by the servomotors mounted in the robotic system, in which the corresponding encoders modulated the hip, knee, and ankle joint kinetics^38^. Specifically, the active force was defined as a positive directional rotation force occurring in line with the target movement direction. In contrast, the resistive force was described as a negative directional rotation force acting against the target movement direction^38^.

The force equation is expressed as ***u*^**_*ext*_ = $\frac{g}{s+g}$ (***ua*** + *g* $\widehat{{\varvec{F}}}$ (***q***)***q*˙** +$\widehat{{\varvec{F}}}$ (***q***) ***q*˙**) − *g* $\widehat{{\varvec{F}}}$ (***q***)***q*˙**.

Clinically, an increase in force on the affected side represented an increase in voluntary strength recovery of the paretic lower extremity. In contrast, a high resistance force indicated opposition that constrained active limb movement during gait.

Furthermore, the kinematic and kinetic computing software of the ICT system was used to examine the ankle, knee, and hip joint stiffness associated with RAGT. Graphical data were analyzed using a maximal sampling rate of 72 Hz (gait cycle varies with the customized preferred walking velocity; frequency range 28–72 Hz at 1.00–2.60 km/h) using a moving averaging filter. The stiffness *k* in the hip-knee joint-segment indicated the resistance provided by an elastic body segment to deformation. Spasticity-related stiffness was computed based on the joint angular displacement and resistive torque data, using a linear regression equation during the gait cycle ^31^, which was expressed as *k*_stiffness_ = $\frac{\mathrm{F}}{\mathrm{ \theta}}$, where *F* is the resistive force acting on the knee, hip, and ankle joints; and *θ* is the angular displacement produced by the force acting on the corresponding joint. In essence, a lower stiffness value (approximately “0 or negative value”) represented a more significant active movement.

### Clinical spasticity assessment

The MAS is a commonly used quantitative measure of spasticity or muscle tone in response to passive limb movements^42^. The ankle, knee, and hip flexors and extensors of the paretic limb were tested according to a standardized procedure^43^. The grading ranged from 0 (“normal tone”) to 4 (“rigid”). The MAS has been reported to be significantly responsive in detecting changes in muscle tone in patients with hemiparetic stroke, and its reliability (weighted kappa = 0.87, standard error = 0.03, *P* < 0.001) has been well established^43^.

The FMA-LE synergy scale (sub-score II index) was used to examine the lower-extremity sensorimotor function and ankle–knee–hip joint function because it represents volitional or voluntary locomotor movement patterns, which include flexor and extensor synergy. The flexor synergistic movement pattern comprised maximal hip flexion (abduction/external rotation), maximal knee flexion, and maximal ankle flexion. In contrast, the extensor synergistic pattern consisted of hip extension/adduction, knee extension, and ankle plantarflexion. Resistance was applied to ensure active movement and to evaluate both movement and strength. The ordinal grading scale consisted of values as follows: 0 “cannot perform,” 1 “can partially perform,” and 2 “can completely perform.” Clinically, 0 and 1 indicate an abnormal movement synergistic pattern, whereas a score of 2 indicates the normal volitional movement synergy constituting the normal locomotor pattern. The total sub-score ranged from 0 to 6 points for the volitional movement with the flexor synergy test and 0 to 8 points for the volitional movement with the extensor synergy test^44^. The reliability and validity of the kinematic and kinetic measurements in ICT system were well established, intraclass correlation coefficient_3,k_ = 0.96, and *r* = 0.65–0.93, respectively^34,38^.

### Intervention

**Figure 3:**
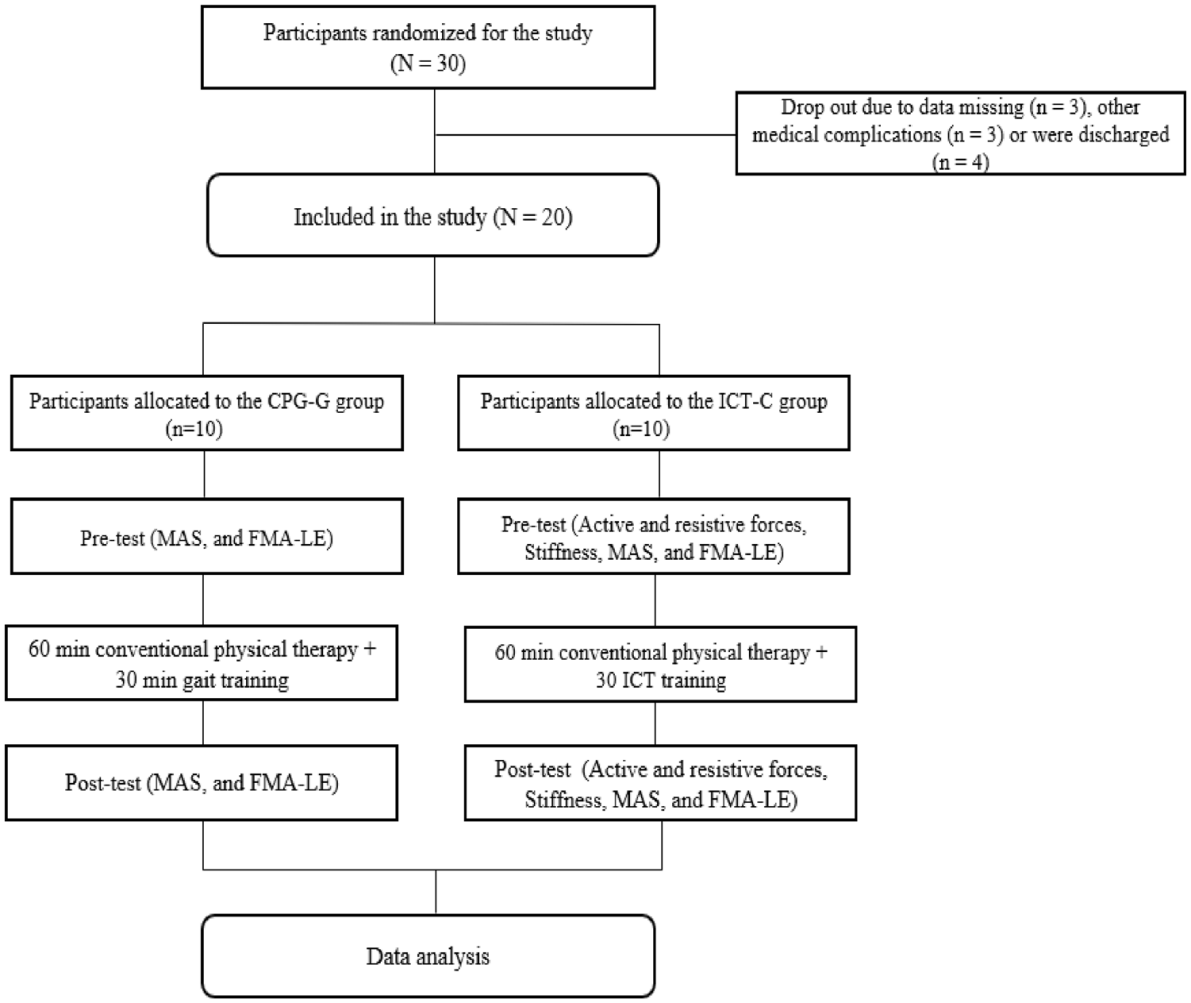
Flow chart. *CPT-G* conventional physical therapy and gait training, *ICT-C* ankle–knee–hip interlimb coordinated humanoid robot combined with conventional physical therapy.

Both groups underwent an additional session of 30 min of therapy daily, 7 days/week, for 2 weeks. The CPT-G group underwent general inpatient treatment, including at least one 60-min session of physical therapy per day. An additional 30-min standard physical therapy session was executed in the pre-ambulatory phase and/or for gait training activities. CPT-G was based on neurodevelopmental approaches and was conducted by skilled and experienced physical therapists. The ICT-C group underwent general physical therapy, which included at least one 60-min physical therapy session and the additional 30-min ICT session. Anthropometric data, including height, weight, foot size, thigh length, shank length, and ankle height, were measured and entered into the participant database. These data were used to automatically adjust the length and optimal gait cycle of the exoskeleton legs according to each participant's conditions. This provided the patients a sense of safety using the suspension vest secured with elastic straps and connected to the harness mounted on the counterweight system. Depending on the initial clinical conditions of the participant (e.g., pain, muscle weakness, spasticity, tolerance, fatigue, or endurance), approximately 40%–60% (adjustable range, 0%–100%) of the total body weight was sustained in the first session, which was gradually reduced by 5%–10% over the sessions. The guidance force mode in the ICT system was used to increase the active engagement during robot-assisted gait retraining accurately. According to the participant's ability to improve beyond the initial target level (e.g., 40 Nm), the ICT system interactively adjusted the walking speed and resistive torque parameters based on patient comfort and preference while attempting to minimize kinematic trajectory errors. The assistance guidance force was systematically reduced from 100% (passive mode) to 0% (active mode). In the active mode, the system could compensate for the weight, resistance, and inertia of the hemiparetic leg to achieve symmetrical, optimal gait patterns. Furthermore, it provided real-time feedback from the treadmill, such as gait kinematics (joint angles), kinetic forces (active, resistive torque, and stiffness) on the ankle–knee–hip interlimb coordinated movement, and active torque on the ankle joint movement. During each session, the patients were provided with constant verbal encouragement based on the results of real-time kinematic and kinetic data to optimize their gait patterns. The ICT system was provided with virtual reality (VR)/augmented reality (AR) games (e.g., a virtual side scrolling game Jordan jumping and taking the coins) and AR scenes (e.g., three-dimensional walking to explore a king’s castle) to maximize the patient’s interest, motivation, and active engagement, while decreasing anxiety and depression during the ICT session (Fig. 3)^26^.

### Statistical analyses

Statistical data were expressed as means (M) and standard deviations (SD). The present preliminary clinical study involved a non-superiority design in which the two-way analysis of variance (ANOVA) and paired *t*-test were performed separately. The two-way analysis of variance (ANOVA) was applied for MAS and FMA-LE data. Significant differences between the control and experimental groups were subjected to Tukey’s post-hoc test. The paired *t*-test was used to compare the biomechanical characteristics (kinetics, kinematics, and stiffness) between pre-ICT and post-ICT in the experimental group. The Chi-square test was used to analyze categorical demographic variables. Continuous variables were analyzed using the Kolmogorov–Smirnov test. Independent *t*-tests were used to compare general characteristics of the patients between the groups. Additionally, Spearman’s rank correlation was used to determine the correlation among the MAS score, FMA-LE, and stiffness. Based on our previous study, a power analysis using G-Power software (G-power software 3.1.9.4; Franz Faul, University of Kiel, Germany) was performed to compute the minimum sample size requirement^31^. The sample size was determined to be 30 based on the effect size (eta squared, *η*^2^ = 0.6) and power (1 − *β* = 0.80) on minimal clinically important difference (MCID) of FMA-LE and from torque and force data^31^. SPSS for Windows (version 25.0; IBM Corp., Armonk, NY, USA) was used, with a significance level set at α = 0.05.

## Results

### Kinematic data

**Figure 4:**
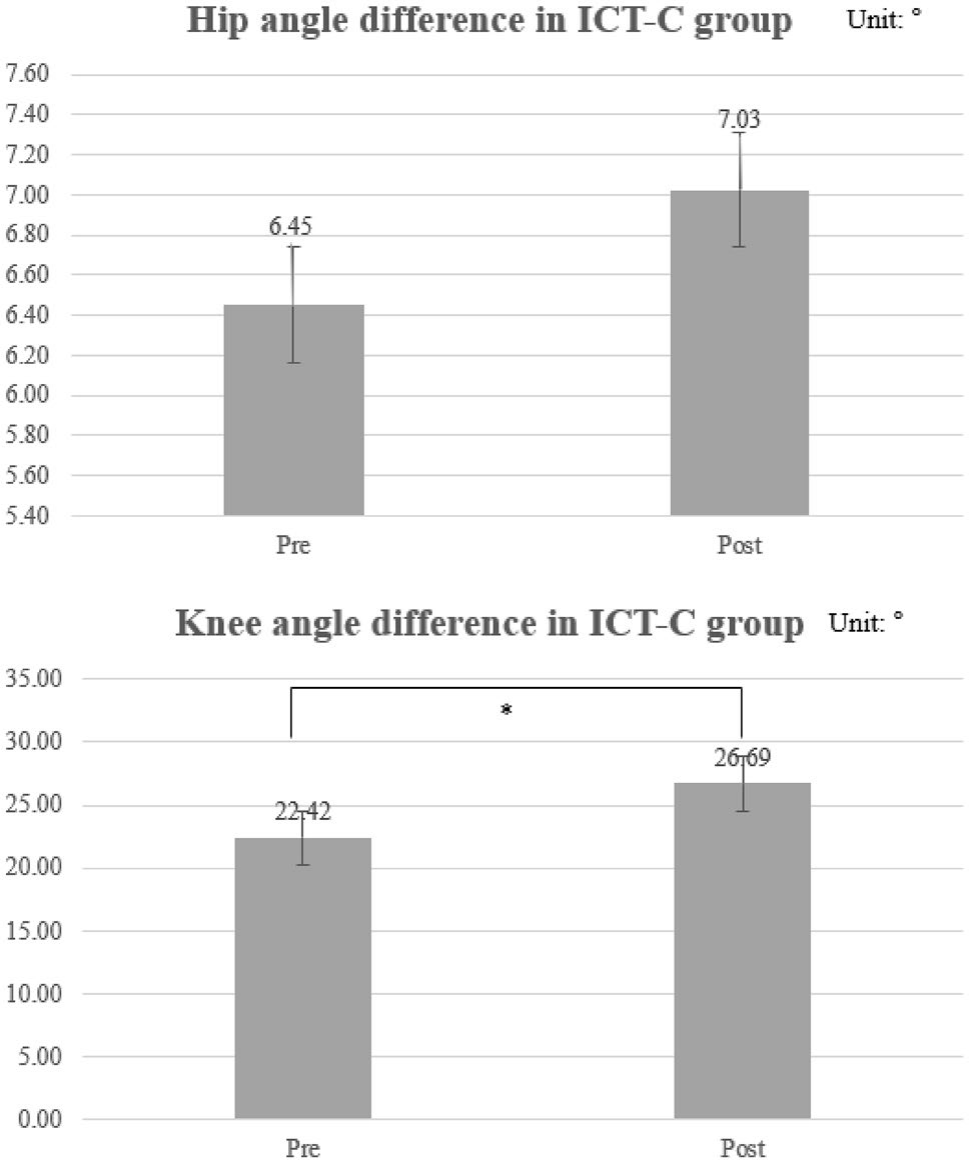
Paretic hip and knee angle kinematics in ICT-C group (unit: degree). *ICT-C* ankle–knee–hip interlimb coordinated humanoid robot combined with conventional physical therapy; *Denotes significance at *P* < 0.05; Number, mean; Bar, standard deviation.

The paired *t*-tests showed that the mean post-ICT knee joint angle (M = 26.69, SD = 1.10) was more increased than the mean pre-ICT knee joint angle (M = 22.42, SD = 0.61; t (9) = − 14.59; *P* = 0.00) in the ICT-C group, indicating improved knee joint movement after ICT-C in patients with hemiparetic stroke (Fig. 4).

### Kinetic data

The paired *t*-tests revealed that the mean post-ICT hip active force (M = 1.32, SD = 0.52; *t* (9) = − 2.56; *P* = 0.03) was significantly greater than the mean pre-ICT hip active force (M = 0.59, SD = 0.48) in the ICT-C group. The paired *t*-tests revealed that the mean post-ICT knee active force (M = 1.66, SD = 1.95; *t* (9) = − 2.47; *P* = 0.04) was significantly greater than the mean pre-ICT knee active force (M = 0.05, SD = 0.04) in the ICT-C group. The paired *t*-tests revealed that the mean post-ICT ankle active force (M = 1.52, SD = 1.06; t (9) = − 2.71; *P* = 0.02) was more increased than the mean pre-ICT ankle active force (M = 0.46, SD = 0.67) in the ICT-C group, indicating an improved hip–knee–ankle joint coordinated force after ICT-C. The standardized effect size index, d, ranged from 0.64 to 0.67, indicating large clinical effects (Table 2).

**Table 2:** Comparison of active force data in the paretic limb in ICT-C (unit: N).

	ICT-C		Mean difference	*t*-value	*P*-value	Effect size
	Pre-test	Post-test				
Hip active force	0.59 ± 0.48	1.32 ± 0.52	0.73	− 2.56	0.03*	0.64
Knee active force	0.05 ± 0.04	1.66 ± 1.95	1.61	− 2.47	0.04*	0.64
Ankle active force	0.46 ± 0.67	1.52 ± 1.06	1.06	− 2.71	0.02*	0.67
Hip resistive force	6.18 ± 0.21	2.08 ± 0.11	− 4.1	61.61	0.02*	1.00
Knee resistive force	1.53 ± 0.80	0.12 ± 0.09	− 1.41	5.19	0.001*	0.87
Ankle resistive force	− 0.84 ± 0.21	− 0.07 ± 0.53	− 0.77	− 4.80	0.02*	0.85

*ICT-C* ankle–knee–hip interlimb coordinated humanoid robot combined with conventional physical therapy; *Denotes significance at *P* < 0.05.

The paired *t*-tests showed that the mean post-ICT hip resistive force (M = 2.08, SD = 0.11; *t* (9) = 61.61; *P* = 0.00) was significantly greater than the mean pre-ICT hip resistive force (M = 6.18, SD = 0.21) in the ICT-C group. The paired *t*-tests indicated that the mean post-ICT knee resistive force (M = 0.12, SD = 0.09; *t* (9) = 5.19; *P* = 0.001) was more increased than the mean pre-ICT knee resistive force (M = 1.53, SD = 0.80) in the ICT-C group. The paired *t*-tests revealed that the mean post-ICT ankle resistive force (M = − 0.07, SD = 0.53; *t* (9) = − 4.80; *P* = 0.001) was significantly greater than the mean pre-ICT ankle resistive force (M = − 0.84, SD = 0.21) in the ICT-C group, indicating an improved hip–knee–ankle joint coordinated force after ICT-C. The standardized effect size index, d, ranged from 0.85 to 1.00, representing large clinical effects (Table 2).

The paired *t*-tests showed that the mean post-ICT hip stiffness (M = 0.72, SD = 0.17; *t* (9) = 1.32; *P* = 0.00) was significantly greater than the mean pre-ICT hip stiffness (M = 1.53, SD = 0.23) in the ICT-C group. The paired *t*-tests revealed that the mean post-ICT (M = 0.70, SD = 0.15; *t* (9) = 7.31; *P* = 0.00) was more increased than the mean pre-ICT knee stiffness (M = 1.17, SD = 0.11) in the ICT-C group. The paired *t*-tests revealed that the mean post-ICT ankle stiffness (M = 0.40, SD = 0.11; *t* (9) = 2.34; *P* = 0.04) was significantly greater than the mean pre-ICT ankle stiffness (M = 0.67, SD = 0.33) in the ICT-C group, indicating an improved ankle, knee, and hip joint coordinated force after ICT-C. Moreover, the standardized effect size index, d, ranged from 0.68 to 0.95, suggesting large clinical effects (Table 3).

**Table 3:** Peak passive stiffness between pre- and post-test in a paretic hip, knee, and ankle stiffness (unit: Nm).

	ICT-C		Mean difference	*t*-value	*P*-value	Effect size
	Pre-test	Post-test				
Hip stiffness	1.53 ± 0.23	0.72 ± 0.17	− 0.81	9.16	0.00*	0.95
Knee stiffness	1.17 ± 0.11	0.70 ± 0.15	− 0.47	7.31	0.00*	0.87
Ankle stiffness	0.67 ± 0.33	0.40 ± 0.11	− 0.27	2.34	0.04*	0.68

*ICT-C* ankle–knee–hip interlimb coordinated humanoid robot combined with conventional physical therapy; *Denotes significance level at *P* < 0.05.

### Clinical spasticity and FMA-LE synergy data

ANOVA showed significant differences in the hip extensor and ankle dorsiflexor MAS scores between the CPT-G and ICT-C groups (*P* = 0.000; 0.043). The post-hoc analysis confirmed more decreased hip extensor and ankle dorsiflexor spasticity after ICT-C than CPT-G, suggesting that patients with hemiparetic stroke had decreased muscle spasticity after ICT-C but not after CPT-G (Table 4).

**Table 4:** Modified Ashworth scale and Fugle-Meyer assessment lower extremity.

	CPT-G		ICT-C		*P*-value			Effect size
	Pre-test	Post-test	Pre-test	Post-test	Time effect	Between groups	Time × group	
Hip flexor	0	0.14 ± 0.38	0.31 ± 0.59	0.13 ± 0.35	0.837	0.077	0.107	0.003
Hip extensor	0	0	0.44 ± 0.62	0.25 ± 0.46	0.335	0.000*	0.335	0.011
Knee flexor	0.14 ± 0.38	0.36 ± 0.63	0.50 ± 0.53	0.25 ± 0.46	0.698	0.555	0.368	0.004
Knee extensor	0.14 ± 0.38	0.29 ± 0.49	0.31 ± 0.60	0.25 ± 0.46	0.678	0.580	0.335	0.005
Ankle dorsiflexor	0.29 ± 0.76	0.31 ± 0.59	0.14 ± 0.38	0	0.635	0.043*	0.527	0.006
Ankle plantarflexor	0.21 ± 0.57	0.14 ± 0.38	0.37 ± 0.74	0.13 ± 0.35	0.187	0.565	0.466	0.009
Flexor synergy	4.50 ± 1.20	5.50 ± 0.53	3.86 ± 1.83	5.29 ± 0.99	0.000*	0.116	0.615	
Extensor synergy	6.50 ± 1.85	7.50 ± 1.07	5.71 ± 2.16	7.07 ± 1.00	0.007*	0.170	0.797	
Total synergy	10.00 ± 1.41	14.00 ± 2.56	9.14 ± 2.25	12.79 ± 2.64	0.000*	0.057	0.513	

*MAS* modified Ashworth scale, *CPT-G* conventional physical therapy and gait training, *ICT-C* ankle–knee–hip interlimb coordinated humanoid robot combined with conventional physical therapy, *FMA-LE* Fugl-Meyer assessment lower extremity; *Denotes significance level at *P* < 0.05.

ANOVA failed to yield a significant difference in the FMA-LE synergy scale score between CPT-G and ICT-C (*P* = 0.12, 0.17; Table 4).

### Correlation among MAS, FMA-LE, and stiffness

A strong correlation was observed between knee stiffness and knee extensor spasticity during gait (*r* = 0.70, *P* = 0.03) in the ICT-C. The correlation between ankle dorsiflexor spasticity and ankle stiffness (*r* = 0.68, *P* = 0.02) and ankle plantar flexor spasticity and ankle stiffness (*r* = 0.60, *P* = 0.04) were moderate. A moderate correlation was also found between hip extensor spasticity and hip stiffness (*r* = 0.28, *P* = 0.04; Table 5). Additionally, the correlation between hip stiffness and knee flexor spasticity was moderate. A moderate negative correlation was observed between flexor synergy and ankle stiffness (*r* = − 0.43, *P* = 0.04). The correlation between knee flexor spasticity and flexor synergy and that between knee extensor and extensor synergy was moderate (*r* = − 0.43, *P* = 0.02; Table 5).

**Table 5:** Spearman’s rank correlation between MAS, stiffness, and FMA synergy.

	MAS spasticity					
Stiffness	Hip flexor	Hip extensor	Knee flexor	Knee extensor	Ankle dorsiflexor	Ankle plantarflexor
Hip	0.459	0575*	0.279*	0.169	0.234	0.041
Knee	0.204	0.124	0.592*	0.697*	0.271	− 0.039
Ankle	− 0.037	− 0.014	0.025	0.168	0.684*	0.600*

	FMA synergy					
	Flexor synergy			Extensor synergy		
Hip	− 0.01			0.01		
Knee	0.16			− 0.18		
Ankle	− 0.42*			− 0.31		

	MAS spasticity					
FMA synergy	Hip flexor	Hip extensor	Knee flexor	Knee extensor	Ankle dorsiflexor	Ankle plantarflexor
Flexor synergy	− 0.14	− 0.04	− 0.43*	0.01	− 0.41	− 0.14
Extensor synergy	0.01	− 0.22	− 0.01	− 0.43*	− 0.27	− 0.07

*MAS* modified Ashworth scale, *FMA* Fugl-Meyer assessment; *Aenotes within-group significance at *P* < 0.05.

## Discussion

To the best of our knowledge, this is the first randomized controlled trial (RCT) on Walkbot-based RAGT to evaluate comparative effects of ICT-C and CPT-G on clinical spasticity and abnormal synergy control in patients with acute hemiparetic stroke and biomechanical effects of Walkbot ICT on kinematic (spatiotemporal parameters and angles) and kinetic (active force, resistive force, and stiffness) gait parameters before and after ICT. As hypothesized, ICT-C demonstrated more positive effects in clinical spasticity and abnormal synergy control than CPT-G alone. ICT was associated with positive effects on both kinematic (spatiotemporal parameters and angles) and kinetic (active force, resistive force, and stiffness) gait parameters. Most importantly, the present robotic interactive gait training provided optimal ankle–knee–hip inter-joint coordinated training, which reduced spasticity and associated stiffness and abnormal synergistic (extensor) gait patterns while improving the active participation and associated active force during gait.

The kinematic analysis demonstrated that mean hip and knee joint angular displacements had improved by 8% and 16%, respectively, as a function of ICT-C in our cohort with acute hemiparetic stroke. Before RAGT, increased compensatory hip hiking and circumduction gait were observed during the swing phase (owing to limited hip triple ankle–knee–hip flexion along with forward momentum). An 11% decrease in ankle dorsiflexion at initial contact, hip extension, and limited ankle plantarflexion was evident at the terminal stance. After the robotic intervention, the lower-extremity extensor synergy pattern accompanying circumduction and hip hiking was substantially diminished because increased hip triple ankle–knee–hip flexion along with forward momentum was observed during the swing phase. In contrast, an 8% increase in ankle dorsiflexion at initial contact, hip extension, and ankle plantarflexion at the terminal stance was remarkably achieved. Conversely, those who exhibited a lower-extremity flexor synergy pattern accompanying gluteus maximus weakness and excessive hip circumduction showed a substantial reduction in this pattern because of the triple interlimb coordination of ankle–knee–hip extension along with the extensor momentum were observed during mid-stance. With a 3% increase in ankle plantarflexion during the pre-swing phase, hip flexion and ankle dorsiflexion during the loading response phase were achieved. Our findings were consistent with previous kinematic studies that reported 3%, 10%, and 15% improvements in hip, knee, and ankle joint angular displacements, respectively, after RAGT in 21 patients with chronic hemiparetic stroke^29^. Bonnyaud et al. reported that RAGT using Lokomat improved the paretic hip (1.3°), knee flexion (1.9°), and ankle plantarflexion (0.6°) range movement compared with conventional gait training after 20 min in 26 patients with hemiparetic stroke^45^. Interestingly, ankle dorsiflexion remained unchanged. Such a lack of ankle dorsiflexion might have resulted from the fact that Lokomat does not have an independent ankle actuator that can help accurately modulate the ankle dorsiflexion-plantarflexion movement in coordination with hip and knee joint movements during ambulation. A significant difference between the Lokomat and Walkbot exoskeletal systems is an independent ankle actuator in Walkbot. The Lokomat system is equipped with hip-knee joint actuators, which provide RAGT focusing on hip and knee joint movements. In contrast, the ICT system comprises ankle–knee–hip joint actuators, which allow the natural ankle–knee–hip interlimb coordinated movement during locomotion^9,26^. Therefore, the underlying rationale for such kinematic improvement may be associated with the fact that RAGT provided corrected gait-specific kinematic (kinesthetic) feedback to the spastic muscles that were synergistically activated into hip and knee extension during the stance phase of walking and hampered the flexion of the hip and knee for foot clearance^29,46^. Little et al. observed that abnormal hip hiking and circumduction affected foot clearance owing to limited hip and knee flexion kinematics compared with limited ankle dorsiflexion kinematics alone. This highlights the importance of ankle–knee–hip inter-joint coordinated locomotor control^47^.

The kinetic analysis revealed substantial enhancements in active force, resistive force, and stiffness in the hip (55%; − 197%; − 113%), knee (97%; − 92%; − 67%), and ankle (70%; − 13%; − 68%) putatively as a function of ICT-C. Notably, gait-related active force gains in the hip, knee, and ankle joints improved by 13%–197% in ICT-C. This finding substantiates previous RAGT evidence using Walkbot, demonstrating more substantial improvements in hip flexion (1.05 Nm) and extension (0.16 Nm) active torques, hip flexion (− 0.56 Nm), and extension (− 0.26 Nm) resistive torques, and hip flexion (− 0.54 Nm/°) and extension (−0.2 Nm/°) resistive stiffness as a function of the intervention in patients with subacute stroke^31^. Notably, such positive changes induced by RAGT in active torque, resistive torque, and associated stiffness ranged from 20 to 80% in patients with chronic stroke who exhibited inherently increased joint stiffness and muscle shortness associated with spasticity despite prolonged deconditioning after stroke onset^31^. Certainly, it is plausible that such paradoxical phenomena between active and resistive forces or stiffness support the commonly held premise of reciprocal inhibition of spastic plantarflexors and associated extensor synergetic gait pattern impairment after stroke^48,49^. The kinematic and kinetic findings reported in the literature support the notion that RAGT using just hip-knee joint, or ankle–foot plate actuators alone cannot mitigate the extensor or flexor synergetic gait impairment in stroke rehabilitation. Close biomechanical coordinated coupling between the ankle, knee, and hip joints is recognized in a healthy gait pattern^41^, whereas exaggerated hip flexion, hiking, or circumduction synergy is commonly used to clear the toe as a compensatory manifestation in a stereotypical hemiparetic gait pattern^10^. Given the dynamic role of the ankle–knee–hip interlimb locomotor coordination, our findings are consistent with those of other studies suggesting that ankle dorsiflexion and plantarflexion torques help in limb advancement^10,50,51^. Recent ankle robotic biomechanical evidence confirmed that ankle robotic assistance helped generating sufficient ankle dorsiflexion and plantarflexion kinematic (7°) and torque values (20 Nm), which play a cardinal role in the paretic limb forward advancement moment (22 Nm) of patients with hemiparetic synergetic gait impairment^3,10,51,52^. In the present robotic paradigm, the ICT detected altered biomechanical characteristics associated with spasticity and synergistic gait patterns that were initially guided and facilitated based on the real-time kinematic and kinetic feedback about the ankle–knee–hip locomotor movement. Such locomotor movement sense feedback is essential for proprioceptive sensory awareness required during the locomotor skill relearning because the majority of the patients with hemiparetic stroke experience altered sensorimotor function following the sensorimotor cortex lesion. The accurate sensory inputs about locomotor related joint angle and force are transmitted to the spinal cord, subcortical level, and cortical level of the sensorimotor cortex via the ascending proprioceptive pathways (dorsal column and medial lemniscus)^53,54^. The locomotor related sensory feedback involves the modulation of the supraspinal networks including subthalamic locomotor region, cerebellar locomotor region, and descending pontine locomotor regions, and mesencephalic locomotor regions, where locomotor signals are transmitted via pontine reticulospinal pathway to the spinal cord central pattern generators, thereby regulating the interlimb coordinated locomotor pattern^55,56^.

Additionally, task-oriented locomotor re-learning was progressively challenged by increasing the amount of active participation or the use of the paretic and nonparetic limbs (1000 repetitions or steps), which were underutilized in the conventional therapeutic approach (292.5 steps)^57^. Furthermore, our clinical FMA-LE and MAS data revealed that the abnormal synergy pattern was significantly reduced along with improvements in spasticity in the ankle and hip joints, as evidenced by increased volitional movement with synergy in FMA-LE. Correlational statistics demonstrated that the hip, knee, and ankle joints' resistive stiffness was moderately related to spasticity in the hip extension, knee extension, ankle dorsiflexion, and ankle plantarflexion muscles and inversely correlated with volitional movement with synergy in FMA-LE (Table 4). These results corroborate the classical relationship between spasticity and stiffness, as well as the synergistic pattern^58^. Ankle spasticity has been consistently reported to be associated with ankle stiffness (*r* = 0.23) and abnormal synergy in adults with spastic hemiparetic gait^59^. To date, no clinical evidence exists regarding the unpinning neuromechanical relationships between spasticity and stiffness, as well as the synergistic pattern during locomotion when RAGT is implemented. It is plausible that enjoyable (VR), active, repetitive locomotor movement (1000 repetitions or steps) via ICT can facilitate agonistic activation (dorsiflexion) while reciprocally inhibiting abnormal spasticity and synergistic antagonist activation (e.g., plantarflexion) during the initial contact of the gait cycle^57^*.* Abnormal spasticity and associated stiffness in the ankle plantar flexors are strongly influenced by stretch reflex hyperexcitability as a result of cortical disinhibition in adults with hemiparetic stroke^59^, which generates a stereotypical asymmetrical extensor synergistic gait pattern^46^. Certainly, such an altered synergistic gait pattern was mitigated by active, enjoyable, repetitive locomotor movement via ICT combined conventional physical therapy, which may be related with neuroplasticity and associated locomotor functional recovery^3,8,26,30^. In fact, our patients’ post-intervention survey reported that the ICT was enjoyable and motivating. The technology acceptance was previously evaluated by conducting the Participant Satisfaction Questionnaire, which yielded that most patients who successfully completed the RAGT reported that the Walkbot RAGT was safe, fun, and beneficial for gait training^26^. Similarly, our previous functional magnetic resonance neuroimaging study validated that locomotor training using VR increased blood oxygenation level-dependent signals (0.6) in the ipsilesional primary sensorimotor cortex in patients with chronic hemiparetic stroke^60^. Limitations of the current research should be considered in future investigations. A major limitation is that although the present results are promising, they should be interpreted carefully when attempting to extrapolate the current findings to clinical practice and to the management of stroke participant rehabilitation due to the small sample size. The initial sample size was proposed to be 30 patients while accounting for the 25% attrition rate. However, due to the complicated nature of patients with acute stroke, only 20 of 30 (66%) patients who successfully completed the pre-test, intervention, and post-test were included in the final data analysis. The remaining patients could not complete the test due to fatigue, other comorbidities, medical complications, or early discharge. Another limitation is that biomechanical data were only obtained from the ICT-C group because biomechanical assessment outcome data may be influenced by the likelihood of the ICT-C group to be more familiar with the Walkbot biomechanical assessment than the CPT-G group. Nevertheless, in future research, it would be more appropriate to implement the biomechanical assessment for both groups after sufficient familiarization of the Walkbot biomechanical assessment.

In conclusion, ICT involving ankle–knee–hip movements together with conventional physical therapy in the acute inpatient phase after stroke was associated with improved biomechanical gait profile and clinical status. Moreover, our correlational statistics indicated that the hip, knee, and ankle joints’ resistive stiffness moderately correlated with spasticity in the hip extension, knee extension, ankle dorsiflexion, and ankle plantarflexion muscles and inversely correlated with volitional movement with synergy in FMA-LE in the ICT-C group. Our results highlight the incorporation of ICT with conventional therapy as a successful intervention for abnormal spasticity, synergistic, and altered biomechanical gait impairments in patients with acute stroke. Most importantly, the Walkbot ICT system allows an autonomous liberty to provide accurate real-time quantitative biomechanical feedback as well as an effective and sustainable ankle–knee–hip interlimb coordinated locomotor training, which could serve as a basis for advanced robotic science and medical research.
